# Framing Young Childrens Oral Health: A Participatory Action Research Project

**DOI:** 10.1371/journal.pone.0161728

**Published:** 2016-08-22

**Authors:** Chimere C. Collins, Laura Villa-Torres, Lattice D. Sams, Leslie P. Zeldin, Kimon Divaris

**Affiliations:** 1 Department of Dental Ecology, School of Dentistry, University of North Carolina-Chapel Hill, Chapel Hill, NC, United States of America; 2 Department of Health Behavior, UNC Gillings School of Global Public Health, University of North Carolina-Chapel Hill, Chapel Hill, NC, United States of America; 3 Oral and Craniofacial Health Sciences, School of Dentistry, University of North Carolina-Chapel Hill, Chapel Hill, NC, United States of America; 4 Department of Pediatric Dentistry, School of Dentistry, University of North Carolina-Chapel Hill, Chapel Hill, NC, United States of America; 5 Department of Epidemiology, UNC Gillings School of Global Public Health, University of North Carolina-Chapel Hill, Chapel Hill, NC, United States of America; Forsyth Institute, UNITED STATES

## Abstract

**Background and Objectives:**

Despite the widespread acknowledgement of the importance of childhood oral health, little progress has been made in preventing early childhood caries. Limited information exists regarding specific daily-life and community-related factors that impede optimal oral hygiene, diet, care, and ultimately oral health for children. We sought to understand what parents of young children consider important and potentially modifiable factors and resources influencing their children’s oral health, within the contexts of the family and the community.

**Methods:**

This qualitative study employed Photovoice among 10 English-speaking parents of infants and toddlers who were clients of an urban WIC clinic in North Carolina. The primary research question was: “What do you consider as important behaviors, as well as family and community resources to prevent cavities among young children?” Five group sessions were conducted and they were recorded, transcribed *verbatim* and analyzed using qualitative research methodology. Inductive analyses were based on analytical summaries, double-coding, and summary matrices and were done using Atlas.ti.7.5.9 software.

**Findings:**

Good oral health was associated with avoidance of problems or restorations for the participants. Financial constraints affected healthy food and beverage choices, as well as access to oral health care. Time constraints and occasional frustration related to children’s oral hygiene emerged as additional barriers. Establishment of rules/routines and commitment to them was a successful strategy to promote their children’s oral health, as well as modeling of older siblings, cooperation among caregivers and peer support. Community programs and organizations, social hubs including playgrounds, grocery stores and social media emerged as promising avenues for gaining support and sharing resources.

**Conclusions:**

Low-income parents of young children are faced with daily life struggles that interfere with oral health and care. Financial constraints are pervasive, but parents identified several strategies involving home care and community agents that can be helpful. Future interventions aimed to improve children’s oral health must take into consideration the role of families and the communities in which they live.

## Introduction

Despite widespread acknowledgment of the importance of childhood oral health, little progress has been made to-date in the prevention of early childhood caries (ECC) [1,2]. Improving children’s oral health has been the focus of concerted efforts by numerous policy, professional and academic bodies and groups; however, ECC persists as the most common chronic childhood disease and continues to affect an increasing number of toddlers and preschoolers in the US [3,4]. Importantly, the disease is marked by pronounced health disparities, with social, economic, and racial minorities carrying the greatest disease burden [1,5–7]. ECC and its restorative treatment often require specialty care and procedures involving advanced behavior guidance modalities such as sedation and general anesthesia; these can have multi-level impacts on the children’s physical and psychosocial health, as well as financial consequences for their families, communities, and the health system [8].

Children’s oral health and care is largely determined by their family environment. The influence of the family on children’s oral health and care is well-documented and parents have been the focus of efforts to prevent ECC development [9–20]. Despite these efforts, oral health is frequently articulated as an important but often neglected aspect of well-care during the first years of life. Delivering accurate and actionable information to parents regarding their children’s oral health is fundamental but can be challenging [21].

While much is known regarding ‘proximal’ oral health behaviors that help prevent ECC (e.g., tooth brushing with fluoride toothpaste, healthy diet, and establishment of a dental home), less is documented regarding specific circumstances and influences on families’ daily lives that interfere with optimal oral health and care. Survey-based studies have reported on factors such as parental education, dental neglect, and inability to access health services which function as barriers to adopting the recommended child oral health-related behaviors [16,22,23]. Our knowledge is limited with regard to specific, daily-life and community-related factors that impede optimal oral hygiene, diet, care, and ultimately oral health for children [24]. Our study sought to address this knowledge gap by gaining insight into what parents themselves consider relevant and influential with regard to their children’s oral health, within the contexts of the family and the community. To answer this question, we specifically worked with a low-income group of parents of infants and toddlers who were clients of an urban Women, Infants and Children (WIC) center in North Carolina.

## Methods

### Ethics statement

Written informed consent was obtained by all participants and the study was approved by the Institutional Review Board at the University of North Carolina-Chapel Hill (approval #15–1085).

### Methodology

We used a participatory approach for this study, using Photovoice and Community mapping as research tools. In general, participatory research revolves around “a process of sequential reflection and action, carried out with and by local people rather than on them”, while the main difference compared to ‘conventional’ research is the location of the power in the research process [25]. Photovoice is a qualitative research methodology wherein participants and researchers use photography as a means of highlighting issues and factors related to a given problem in the context of participants’ daily lives and the communities they live in. The sharing and discussing of participants’ own photographs is a powerful means of communicating life experiences, expertise and knowledge [26]. Photovoice has been used in recent studies seeking to understand complex health and social issues including food insecurity, childhood obesity and sexual and reproductive health [27–31]. Results from these studies illustrated how the participatory research approaches can improve the understanding of human needs and social determinants of health. Such information can be used to inform community leaders and decision makers on how to improve health and welfare programs and policies. Community mapping is a method of data collection that allows participants to identify their community resources and assets. This approach can help identify and clarify the concept of community, show how people connect to their community and recognize what places are being or can be used by the community for health promotion [32].

### Participant recruitment and eligibility

In August of 2015, we recruited a group of 10 parents (2 males and 8 females) of infants and toddlers who were clients of an urban WIC center in central North Carolina to participate in this study. Screening and enrollment was conducted by the first author approaching families in the waiting area of the WIC center. At that time, the investigator explained the study objectives, procedures and anticipated schedule of events. The inclusion criterion for this study was that participants should be able to speak and understand English, were parents or caregivers of infants and/or toddlers ages 6–36 months, and clients of WIC. Eligible participants who expressed interest in participating provided their contact information and assent for being contacted when study activities were to begin.

### Research activities

We conducted 5 photovoice sessions for this study. We deemed that this number of sessions provided a balance of time to enact richness and depth of ideas and information with practicality and subject retention. During the first session, participants were familiarized with the study goals and procedures, and were given specific guidance on the use of the digital cameras, best practices and ethical considerations when taking photos. They were instructed to take photos according to each week’s research question without specific guidance on the number of photos to be taken. The research team drafted *a priori* several research questions that became the four photo assignments that participants would answer via the photos. After each session, we provided participants with an assignment which they had one week to complete (i.e., the first assignment was given after the end of the first session and its discussion took place at the second session). The assignments consisted of each participant taking photos based on a specific research question. We repeated these steps throughout the project for a total of four assignments (Table 1).

**Table 1:** Photo assignments and corresponding research questions.

Assignment	Research questions
#1	What is good oral health for you? How is your child’s oral health different from yours?
#2	What series of events lead a child to experience oral health problems?
#3	What do you as parents consider important behaviors to prevent oral health problems among your children? What can you do as parents to prevent oral health problems among your children?
#4	What are family and community resources needed to address the oral health issues of your children? What support do you need as parents to keep your children’s oral health in good shape?

Photos obtained by the participants were compiled and reviewed by them, as a group. The selection of photographs to be discussed in each session was determined by the participants themselves, after browsing through all photographs, with typically two investigators functioning as facilitators of this process. The selected photos were discussed in subsequent sessions according to the SHOWeD process, described by Wang [26]: “What do you **S****ee** here? What is really **H****appening** here? How does this relate to **O****ur** lives? **W****hy** does this situation, concern or strength **E****xist**? What can we **D****o** about it?” After the end of session 4, participants were asked to draw and then discuss a community map, in order to help them prepare for the last assignment. The instructions for this activity were to draw a map of the places they frequently attended during a regular week and weekend, including work, recreation, or household-keeping related places (e.g., grocery shopping).

Breakfast was provided to participants at each session. For this study, participants received a $15 gift card as compensation for the time they contributed to. The duration of the sessions ranged between 77’ and 155’, and they were all held at the local WIC center. All participants contributed to the given assignments and discussion; 6 out of 10 participants attended all 5 sessions. Digital cameras were also distributed to participants for the duration of the study to be used for the photo assignments. Each session -except for the second- was attended by two moderators with knowledge in oral health (first author, a dental hygienist) and photovoice, public health and health behavior (second author, a sociologist). During the fourth session, KD (the senior author, a board-certified Pediatric Dentist with public health training) attended the group discussions and was made available to answer participating families’ questions related to oral health after the end of the group session.

### Analytical approach

Group discussions were digitally recorded and transcribed *verbatim*. Inductive analyses were conducted using analytical summaries, codebook development, double-coding by two investigators (the first and the last author) and summary matrices. The codebook included eight main (“parent”) and 41 sub-codes (“child”) emerged from the data. The 8 parent codes were “child’s diet”, “child’s oral health care”, “child’s oral hygiene”, “community resources”, “consequences of oral disease”, “misconceptions”, “strategies”, and “struggles of daily life”. We followed published recommendations for appropriate reporting of our study findings, as reported in the consolidated criteria for reporting qualitative research [33]. All analyses were carried out using Atlas.ti.7.5.9 software.

## Results

The major themes emerging from each photo assignment are outlined in Table 2 and presented in more detail below. The results are presented by photo assignment, although the quotes presented were drawn from different sessions, given the iterative nature of the discussion of some of the salient topics throughout the 5 photovoice sessions. During analysis, we added a fifth research question, regarding barriers to access oral health services that was purposively left out in the original set of questions, but that came up as a salient theme throughout the project.

**Table 2:** Major (“parent”) themes emerging from the analysis of the Photovoice sessions according to each of the 5 research questions.

Assignment	Research questions	Major emerging themes
#1	What is good oral health for you? How is your child’s oral health different from yours?	Avoidance of problems or costly restorations Psychological distress Pain and suffering Esthetics Oral health problems, such as cavities, trauma, esthetics, and dental restorations Misconceptions
#2	What series of events lead a child to experience oral health problems?	Financial constraints to get healthy food Lack of knowledge regarding oral hygiene, fluoride toothpaste, community water, snacks and beverages, feeding practices, fluoride/toothpaste and pacifiers. Convenience Daily struggles of life (time constraints, disagreements between caregivers) Frustration and difficulty with oral hygiene
#3	What do you as parents consider important behaviors to prevent oral health problems among your children? What can you do as parents to prevent oral health problems among your children?	Oral hygiene, fluoride exposure Healthy diet (beverages, snacks, drink water), feeding patterns Going to the dentist Rules/routines and commitment Modeling older siblings or parents Cooperation among caregivers
#4	What are family and community resources needed to address the oral health issues of your children? What support do you need as parents to keep your children’s oral health in good shape?	Community programs (e.g., WIC) and public insurance Friends, family and social circle Community-based organizations (e.g., churches, community centers, libraries) Schools Healthcare providers beyond dentists (e.g., nutritionists, nurses, pediatricians) Publicly available high-quality information (e.g., WebMD) Information dissemination via community and social hubs such as playgrounds, grocery stores & social media
*	What prevents families from accessing children’s oral health care?	Finances Time/schedules Location Insurance Prioritization of medical over dental care

* denotes the 5^th^ added research question.

### Photo assignment 1

What is good oral health for you? How is your child’s oral health different from yours?

The participants acknowledged the importance of oral health for themselves and their young children. Oral health was mainly conceived as avoidance of problems and costly restorations and one participant remarked that positive oral health behavior is essentially whatever “*keeps you out of the dentist's chair*.” Participants shared personal as well as others’ experiences of children having to undergo restorative dental treatment at a young age due to cavities or trauma. These experiences were accompanied by negative connotations including pain and suffering. Participants also discussed how they believed their own and their children’s oral health and care differed, including -for example- the type of toothpaste they would use, or the perceived importance of “baby teeth”, as we can observe in this short dialogue:

Participant: *They can rot out*, *but that can have a negative effect on*…

Participant: *Their gums and the teeth behind*.

This discussion thread led to additional points regarding the importance of primary teeth, as well as children’s exposure to fluoride and the use of electric versus manual toothbrushes. The participants discussed risks and benefits associated with their children drinking bottled versus community water, and some were unclear about the fluoride content and its role, as shown in the following dialogue:

Participant: *Can't you over*… *I don't know what the word is*? *Fluorinate*?

Moderator: *Fluoridate*.

Participant: *Yourself*.

Participant: *You can but it's a huge amount*. *Like over years*.

Participant: *So tap is better than bottle*.

Participant: *In terms of fluoride*, *yes*.

### Photo assignment 2

*What series of events lead a child to experience oral health problems*? *What prevents families from accessing children’s oral health care*? (Emerging theme)

The participants most frequently reported lack of knowledge as being the major contributor to oral health problems among young children. The knowledge domains were: oral hygiene, including use of fluoridated toothpaste; diet, including types of solid foods; beverages, including community water *versus* bottled, sweetened *vs*. unsweetened beverage; types of snacks; and when children should transition from a bottle to a cup. The overwhelming influence of financial constraints to purchase healthy food was evident:

Participant: *The things that are healthy*, *as far as the juices we were talking about with foods*, *are more expensive the better they are for your child*. *It makes no sense to me*.

Participant: *We live in a country that subsidizes processed food*, *and makes the really good stuff expensive (*…*)*

Another participant shared her experience and a different motivation for (not) purchasing bottled water over other beverages when outside the house.

Participant: *Yeah*, *it's like that same price*, *and at that point I'm like*, *"Well*, *you know*, *I'd rather pay for something tasty*.*"*

Participants articulated budget constraints also in the context of accessing dental services.

Participant: *Like*, *"Oh you have a cavity*. *There's another $100*.*00*. *Oh look*, *there's this issue*.*" It seems like there's always something*. *Oh sure that's covered*, *but you still have to pay for this*.

Participant: *I think that definitely drives people away from going to the dentist …*

Participant: *They figure*, *"As long as I brush my teeth at least once a day*, *try to eat okay*, *then that's how we're going to have to do it because we can't afford to go to the dentist*.*"*

Others parents had to make a decision of prioritizing their children’s health (including their oral health) over theirs, as expressed by the following participants:

Participant: […] *Well he hasn't been to the dentist yet*, *but he's also not all that cooperative*. *My kid's*, *definitely*. *They are up to date on their pediatrician visits*, *my daughter's dental care*. *He'll get there eventually*. *I kind of budget that*, *or I prioritize that*. *I shuffle to make that happen over mine*.

Participant: *I have a tooth*, *it's just bad because it needed a crown*. *I had a root canal and it needed a crown and that's $500*.*00 after insurance*. *That like my food budget for a month*. *That's a month of food for our family*.

**Fig 1:**
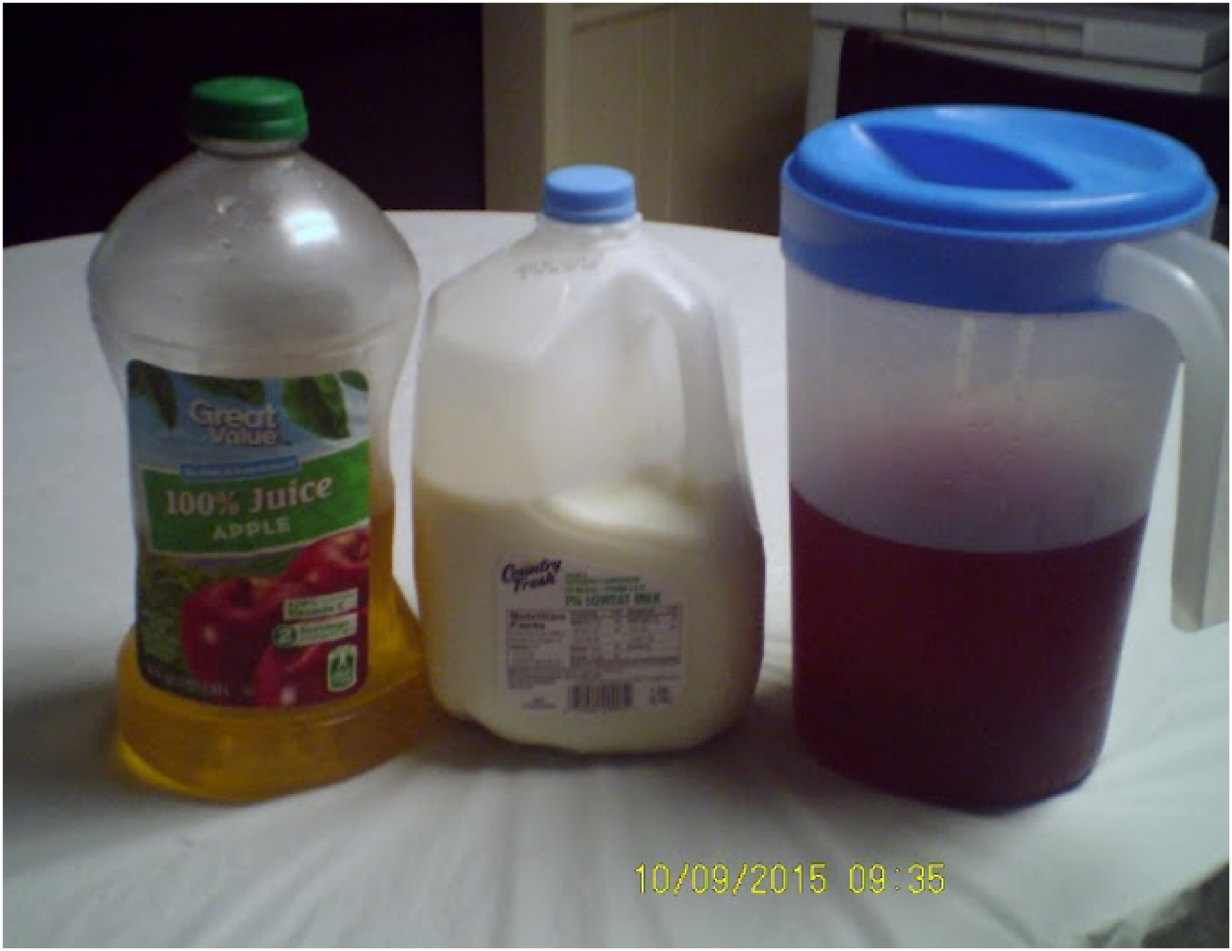
Photograph discussed during the 2^nd^ Photovoice session. Photo of a household’s available beverage options, discussed in the contexts of finances and convenience.

Other daily life struggles were also frequently brought up by the participants. Time constraints appeared to hamper families’ healthy diet and feeding choices. For example, discussion on food and beverage choices revolving around Photo 1 (Fig 1) illustrated the available beverage options in the household. Convenience appeared to underlie most decision-making related to daily choices.

Participant: *Sometimes we as adults do what's quick*. *Not necessarily what's best for you*, *but just what's quick*.

Participant: […] *You're like*, *"Gosh*. *I have to get dinner on the table in an hour*. *Nothings ready*, *nothings thawed*, *nothings prepped*.*" Some of those foods that require more work cost more*. *They cost less*, *but then there's the time fits into the equation*. *If you don't have time*, *then you're stopping and buying a pizza*, *doing those sorts of things*. […]

**Fig 2:**
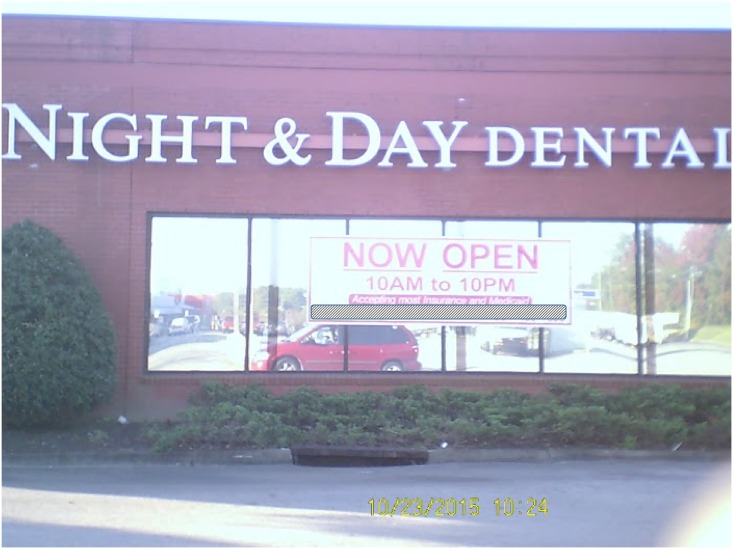
Photograph discussed during the 2^nd^ Photovoice session. Photo of a local dental clinic offering extended open hours, a feature that was considered favorable by participants in terms of access amidst their busy work schedules.

Another daily struggle affecting optimal child oral health care was the inability to take sufficient time off work for routine dental checkups. Participants paired this with inconvenient dental office open times relative to their own work schedule, a discussion thread that was stimulated by another participant photo (Fig 2).

Participant: *I definitely needed a dentist that's open after 5*:*00PM*

Participant: *That's the point*. *To be able to go after school or get the kids to bed and go*.

Participant: *Most offices close at 5*:*00PM or they stop taking appointments at 4*:*30PM*. *It's hard*. *I know I need to go*. *I want to go (*…*)*

Participant: *If you have a half day you're available*, *they're booked for 6 months*.

Participant: *Certain days of the week*.

Participant: *Yep*, *that's another issue*.

In addition, parents did not hide their frustration when trying to enforce children’s daily oral hygiene-related routines.

Participant: *For myself*, *with my daughter*, *in the beginning when we really first started brushing it was really hard*. *I used to get really frustrated*, *and sometimes*… *There'd be days where I'm like*, *"You know what*? *Time to go to bed*.*"*

Participant: *My son’s the opposite*. *He's 6 and he hates routines*. *He hates doing anything that involves going to bed*, *but he also have behavior issues*. *He has ADHD an all the fun stuff*. *So*… *Even though he's used to it*, *and he knows its coming he will fight to no end to not do it*, *and he's the one that's pointing his finger and is like*, *"No*. *I don't want to do that*.*"*

Discordance in parents’ approaches or strategies was also a source of discontent, that stretched thin the patience and engagement in oral health routines.

Participant: *The tension*, *maybe*, *among parents*. *One parent like this and then the other and you're trying to*…

Participant: *My case*, *I'm the one that always brings it up*. *My husband is*… *My kids have him wrapped around their little fingers*. *"Daddy*, *can I have this*?*" "Yes*.*" They're spoiled brats when it comes to daddy*.

Finally, one participant expressed her concern that children may not be able to escape a trajectory of negative (oral) health behaviors or outcomes that the parents have experienced.

Participant: *The children are a reflection of us*. *Sadly*.

### Photo assignment 3

What do you as parents consider important behaviors to prevent oral health problems among your children? What can you do as parents to prevent oral health problems among your children?

**Fig 3:**
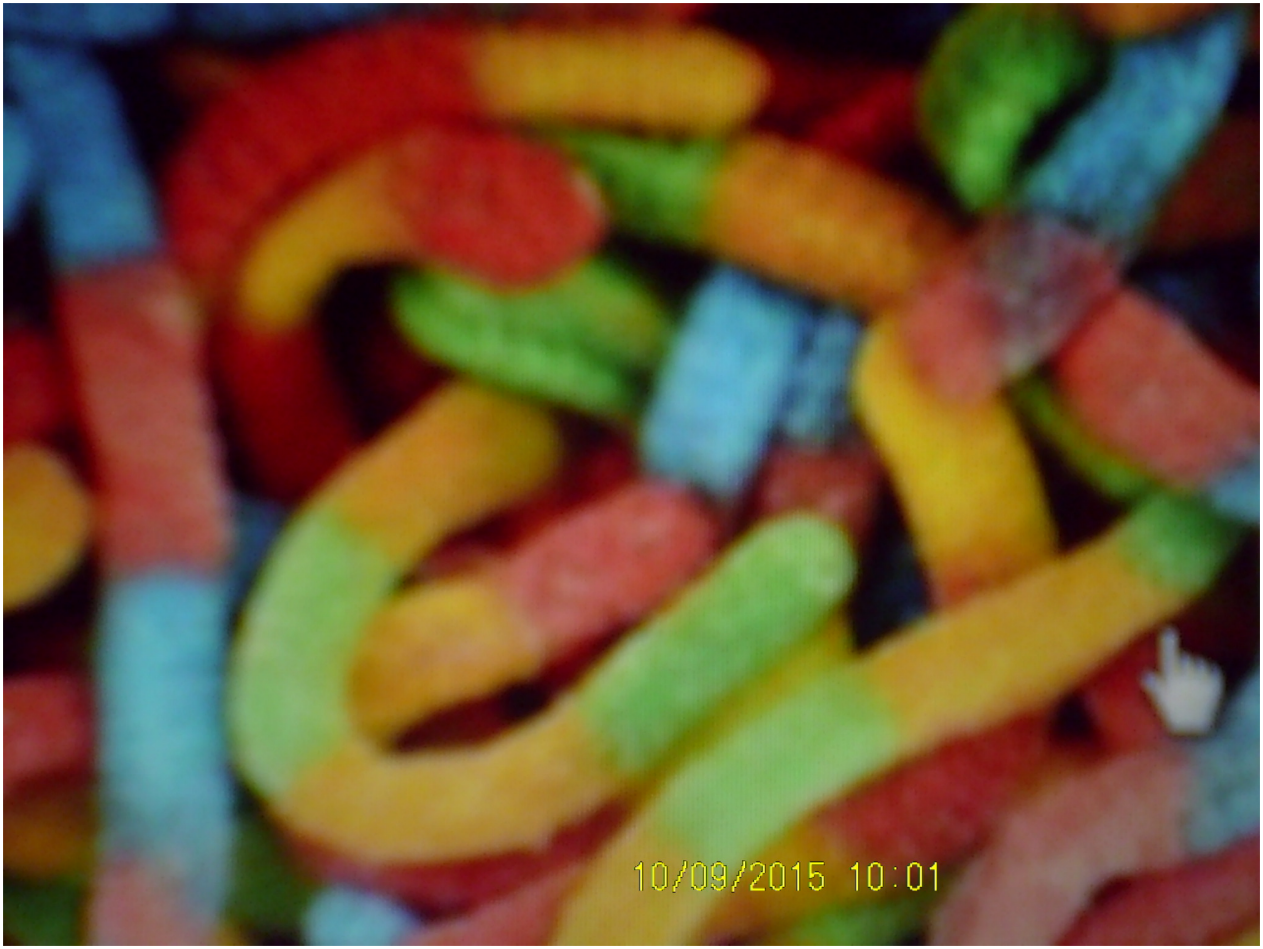
Photograph discussed during the 3^rd^ Photovoice session. Photo of gummy candies which were discussed in the context of deleterious foods or detrimental child oral health-related behaviors that can be avoided.

The participants identified several promising strategies to help promote optimal oral health for themselves and their young children including adherence to professional recommendations regarding oral hygiene (e.g., brushing with fluoridated tooth paste), a healthy, balanced diet (Fig 3) and appropriate oral health care (e.g., preventive dental visits).

Participant: *Okay*. *I think for me good oral health is just make sure I'm brushing my teeth and flossing regularly to prevent any [inaudible]*, *diseases*, *orally with diseases*, *and malfunction*. […]

Participant: *Eating the right foods*.

Moderator: *Eating the right foods*. *What else*?

Participant: *Regular dental exams*.

The parents were also adamant about forming routines with their children, setting and abiding by rules, and cooperating with each other to encourage children’s formation of healthy habits.

Participant: *My son actually has a routine with my father (child’s grandfather)*. *They brush their teeth together*, *because when I try he*… *I try to let him do it*, *he just take the toothbrush*, *and take off running*. *So*, *I just let him*. *Go ahead and y'all go brush your teeth*. *I don't know what they do in there*.

Participant: *Start your day off with a healthy start*.

Participant: *Routine*.

Participant: *Establishing good habits*.

The participants were concordant in that children generally like to have fun when learning new routines and that they should be thinking of novel, innovative ways to encourage healthy oral care habits/routines, including modeling of older siblings or parents. Routines also appear to help parents to deal with their children’s challenging behavior.

Participant: *As being a parent I try and get in a routine*, *because now she'll even*… *I say*, *"Let's go upstairs*. *Get ready for bed"*, *and then she'll say*, *"Brush teeth*? *Brush teeth*?*" It's like now she knows we brush our teeth before we go to bed*. *Like I said*, *getting that routine*, *and doing it with them*. *I think it really helps kind of get them started on their own oral health care routine*.

Moderator: *So maybe slowly incorporating into their routine*.

Participant: *I said*, *"It's all about repetition*.*" You got to just do it over*, *and over*, *and over*.

Moderator: *Do you think that that helps you as a mother*?

Participant: *Yeah it definitely does*, *because*… *I mean*, *if she wants to do it*, *and it's like*… *Like now*, *I don't have to put up as much of a fight with something*. *You know*, *"Okay*, *time to brush teeth*.*" It's something that's kind of fun for her I think*.

### Assignment 4

What are important family and community resources to address the oral health issues of your children? What support do you need as parents to keep your children’s oral health in good shape?

**Fig 4:**
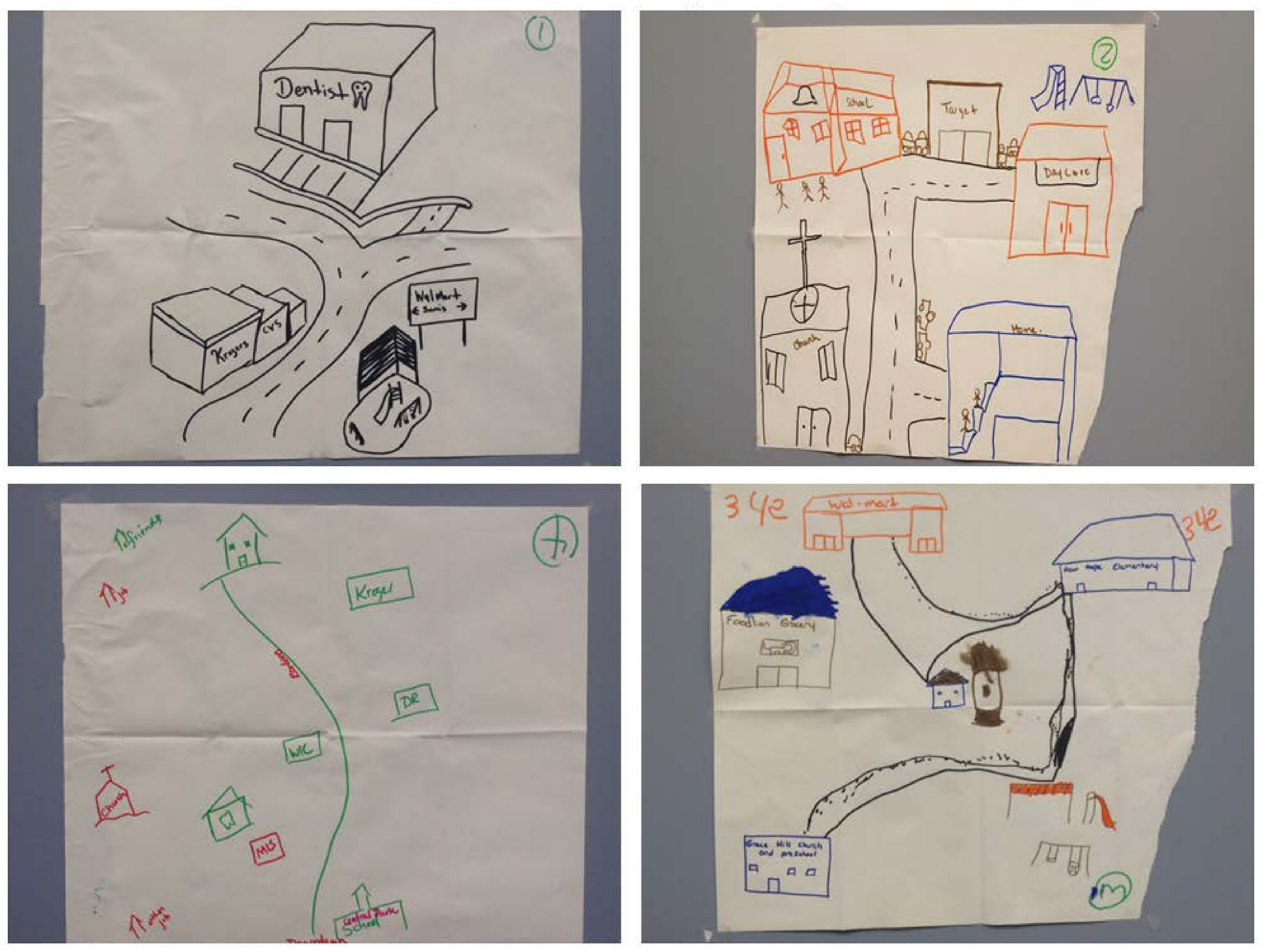
Community maps drawn by participants after the end of the 4^h^ Photovoice session. The participants depicted grocery stores, playgrounds, churches, schools and health care settings along main roads in their communities.

The community maps (Fig 4) were produced at the end of the session prior to assignment 4, to help participants conceptualize their community and the resources available for them. The community maps entailed grocery stores, parks, schools, pharmacies, churches, and health care professionals including dentists. The maps suggested that the participants knew their community well, and did not necessarily consider elements beyond its boundaries as far as daily life was concerned.

The importance of peer support, social networks and community programs relative to participants’ daily lives in general, and specifically with regard to their children’s oral health, emerged prominently in this assignment. As had already been brought up with the community maps, the WIC center, friends, family, churches, community centers, healthcare providers, and other parents formed the parents’ network. Parents also talk about the importance and challenges of creating a trustworthy social network that can help with the care of their children:

Participant: *My son's school*. *He goes to the church preschool*, *so I meet the other parents there*. *Playgrounds*, *libraries*, *communities*. *You meet new faces*.

Participant: *It takes years to gain a network*. *We've been here in* [name of town] *just for this past year*. *We are just now getting into the community and making the friends and talking to them and playing with their kids and having play dates*. *Not leave my kid with them*. *I will go with them and have my kids with them so I can watch my own kids*. *It's not the same as if I was back home up in* [home state]. *I know those people*, *I grew up with those people*. *It's going to take some time*. *It's going to take a couple of years to establish a network here now*.

The participants highlighted the importance of existing community resources including individuals and agencies available to support parents and suggested that some community members may need encouragement or direct help to be linked with and benefit from these resources. In terms of community resources, in addition to WIC, parents suggested that community health fairs, churches, parks and schools are good avenues for oral health promotion for this age group.

Participant: *Like she said*, *educate*, *encourage breastfeeding*. *If you need help*. *Or you need support*, *go seek it*. *Don't just give up on yourself or let if frustrate you*. *Seek help when you need it because it's a lot of help out there for moms that want to breastfeed*.

Participant: *Make sure the mom is able to get the proper nutrient items available to her*.

Participant: *So I put them in touch with individuals or agencies that can help them get the nourishment that they may need*.

Participants voiced concerns regarding the high prices of “healthy food” items for their children and families; although assistance received via WIC is greatly appreciated, their actual purchasing power is limited. WIC generally provides families with dry food such as beans, rice, peanut butter, bread, fruit juice, milk, eggs, tuna, and cheese. More recently, WIC has begun to give out fresh food vouchers, although these are for only $8 per month. This appears to limit families to small amounts of fresh fruits and vegetables for their children.

Participant: *What I like to see is that WIC*, *and agencies like WIC*, *lean more toward fresh foods*. *Like when my*… *When she was pregnant*, *and when she was a newborn*, *we gave out more fresh food vouchers for the mother though*. *Then*, *as the child got older*, *they cut back on that*. *Then they pushed the food for her towards more pre-packaged vegetables*, *and if you look at it*, *the fresh fruit and fresh vegetables would benefit the child even more*. *So*, *keeping the same amount of those throughout the child's history with WIC would work*.

Participant: *The obesity issues*. *The problems that we're seeing*. *The growing rates of type 2 diabetes in children*, *because it's cheaper to get juice*. *You know*, *they give us juice on WIC*. *They don't give us*… *We get the voucher that we can use however we want for fresh fruits and vegetables*, *but 8 bucks*…

Tangentially, participants raised the issue that the contemporary performance-oriented school system does not play a major role in shaping or positively influencing children’s health. For other parents, school was still an agent able to optimize (oral) health.

Participant: […] *When she first started school*, *not here*, *but the other county we were in at*, *I believe it was kindergarten I want to say they did this*, *they had the dentist's and dental hygienists come to the school and talk to them about oral health problems*. *They actually sent a form home for the kids to have fluoride put on their teeth*. *I had that done*.

Other resources and promising community venues for obtaining information and peer support were health or dental fairs and playgrounds; the latter, however, entail risks.

Participant: *(*…*) I noticed that the playground is a good place to talk*, *but it's also a good place where kids get hurt*. *My daughter has chipped two teeth and a busted lip on the playground*. *Now*, *she's going to the dentist constantly to get that filling on those these that she has chipped from being on the playground*.

## Discussion

This study among parents of young children who were clients of an urban WIC center in North Carolina provided informative insights into what parents themselves consider influential community agents and important strategies to promote young children’s oral health. The parents expressed their views and experiences candidly, and offered several useful strategies that could be used in the contexts of oral health education and community intervention. At the same time, our findings illustrated the daily life issues that hamper optimal oral hygiene and health care among this group of families, with financial and time constraints, access to care issues, and gaps in knowledge or misconceptions being the most prominent. Taken together, these findings offer a rich knowledge base that can be utilized for future studies, interventions, or community action programs.

The importance of the family environment in shaping oral health cannot be ignored. Multiple lines of evidence support associations of children’s oral health with their parents’/caregivers’ characteristics including but not limited to socioeconomic status, education level, oral health knowledge, beliefs and attitudes [14–16,19,34]. Parents are in a position to influence and facilitate oral health behaviors in their children because the latter are totally dependent on them for all of their oral health oversight; especially for preschool children, caregivers are responsible for “high stakes” health behaviors such as diet and feeding patterns, oral hygiene and oral health care. All participants acknowledged the importance of their young children’s oral health and their role in it, but most admitted that everyday life circumstances may prevent them from actualizing their positive intentions. They appeared to understand the connection between their own and their children’s oral health, and they tended to prioritize their children’s health care over their own; which is in agreement with previous evidence from qualitative research [35]. Their frequently-articulated frustration with competing priorities and lack of resources or time is indicative of the overwhelming influence of upstream factors and social determinants of health, such as social capital and economic resources [36,37].

It is now understood that location and community (as defined by state, zip code, or neighborhood) is a major determinant of children’s health outcomes, including oral health [38]. The study participants tended to rely on their local community’s available resources for two of the most influential parameters for oral health, namely nutrition (e.g., local grocery stores) and oral health care (e.g., nearby dental practices), as well as social support programs, such as WIC. Ample evidence exists on the multiple mechanisms linking the social context with children’s oral health [39–45], and specific community and neighborhood effects such as location is associated with the development of ECC [46–48]. Little information is available in the literature regarding specific, actionable items or the potential relevance and acceptability of postulated community interventions to improve children’s oral health. Our results suggest that such interventions must be based on a comprehensive understanding of each community, its structure and constituents as well as influential agents and places. Moreover, although large-scale public health interventions (i.e., community water fluoridation) are very effective in both improving oral health outcomes and alleviating health disparities, it is evident that local and personal factors, such as misconceptions about its intent and safety, may dilute the end-point benefit of the intervention, particularly among those who would most benefit from it. This would also apply to health promotion campaigns aiming to increase early preventive dental visits among very young children, wherein families’ valuation of oral health and the importance of primary teeth may be a major determinant of success.

Another important finding from our study is that several constrains and barriers to achieving optimal child oral health are the time constraints, frustration and exhaustion from the amount of time they have to take care of their children, the lack of immediate support networks, and the lack of affordable day care. Low-income families have to constantly juggle the allocation of limited resources, time for rest, recreation, and own health care, and this appears to impact the time they are willing to or capable of investing in child care. There is strong reasoning in support of links between the provision of affordable day care and the development of health and wellness across the life course, especially for resource-limited families [49,50]. It was evident in conversations in this study that parents would benefit from having down time to rest and attend to other household and personal issues, or even to find other jobs that can increase the income of the household.

The WIC program has long been considered as an important vehicle for promoting young children’s oral health, increasing families’ knowledge and access to care [35,51,52]. Recent evidence from a survey among WIC staff in Florida showed [53] that while the majority were “knowledgeable about the role of caregiver in cleaning the child’s teeth” and the importance of dietary factors, they did not routinely provide oral health counseling or referrals for establishment of a dental home. Although variation in counseling practices between staff within and between WIC centers is to some degree expected these data demonstrate the opportunity that exists for WIC and other federal support programs to become positive agents of oral health promotion for young children [54]. This study’s participants acknowledged the material assistance and overall support that they are receiving from WIC while manifesting the arguably impossible task of following an optimal diet (e.g., rich in fresh fruit and vegetables) using the available resources; because, healthy food is expensive. This issue is important and far-reaching if one considers additional domains beyond poor oral health, such as childhood obesity and malnutrition. Farm-to-preschool programs that subsidize the supply of preschools (and potentially, low-income families) with produce from local producers, like the urban “Farm-to-Preschool and Families” in Springfield, Massachusetts [55], would appear as a meaningful aid both for families, children and small-scale local economies.

The participants identified promising strategies and possible community resources available to improve young children’s oral health and care. For example, modeling of oral hygiene behaviors with an older sibling, which is consistent with classic recommendations of behavior modification for pediatric dental patients [56]. They suggested that establishing oral hygiene routine rules and sticking to them, as well as planning ahead their groceries and meals for the week would be helpful strategies. Additionally, some mentioned the need to bring back school-based oral health prevention services, typically offered by school-assigned dental hygienists and lamented the changing character of contemporary schools that tend to be performance- and testing-driven rather than health-building. These parent-generated suggestions are noteworthy and parallel those emanating from recent qualitative studies among parents of young children in the Netherlands [57] and the United Kingdom [58].

This study’s findings should be regarded in view of its limitations. First, our sample was from one urban WIC center in North Carolina and was limited to English-speaking participants. Other ethnicities or rural families may have different experiences and views regarding their children’s oral health and care. In spite of this, it is likely that several themes and issues identified here (for example financial constraints, cooperation among caregivers, and convenience) may be applicable to other communities. We conducted 5 group sessions and as expected had some participant attrition. Larger numbers (up to 14) of sessions have been recommended for Photovoice [59]. In spite of this, we support that the combined numbers of sessions and participants allowed the investigation to reach a great depth and comprehensively cover the examined topic. Moreover, although our analytical approach was an inductive one, we acknowledge that some themes may have been introduced via the posing of the research questions. For example, the question “What do you as parents consider important behaviors to prevent oral health problems among your children?” may have introduced the notion of “positive oral health-related behaviors” and other questions may have suggested that dental problems are preventable. We believe that this issue did not substantially affect our inference and novel insights.

Finally, Photovoice itself as a methodology entails shortcomings. Evans-Agnew and Rosemberg [60] very recently conducted a critical review of Photovoice-based research and questioned the fidelity of transferring “participants’ voices” into the peer-reviewed literature. Nonetheless, we support that our study greatly benefits from providing insights into real everyday life experiences and community factors that influence children’s oral health, emerging from the participants themselves. Arguably, this type of participatory research tends to be more engaging for the study participants than typical questionnaire-based investigations and this may have prompted the participating families to share experiences and identified issues and strategies that would be virtually impossible to obtain from a survey-type study. Arguably, the “observer effect”, wherein participants modify their behaviors due to being under observation (otherwise termed Hawthorne effect, or reactivity), is present in this type of research. However, it is viewed in the context of a bi-directional researcher-participant interaction; in fact, this interaction, along with reflexivity (the influence a researcher bring to the research process) are inherent characteristics of qualitative research, and are considered strengths relative to quantitative methods [61,62].

Improving children’s, families’ and communities health (including oral health) obviously requires concerted efforts of multiple stakeholders; for this reason, measurable and sustainable improvements in children’s oral health will also require the integration of dental education with other health care and social disciplines [63]. Besides the engagement of the dental and allied health professions, future interventions aimed to improve children’s oral health must take into consideration the reality of families and the communities they live in, realizing that they are major influences of oral and general health promotion, including access to healthy food, health care, social support networks and even day care.

In sum, low-income parents of young children acknowledge the importance of early childhood oral health and understand that they have a pivotal role in determining their oral health trajectory. However, they are faced with daily life struggles that appear to interfere with optimal oral health and care for their young children. Financial constraints are pervasive, but parents identified several mitigating strategies involving home care, planning and community agents that can be helpful. Future interventions aimed to improve children’s oral health must take into consideration the role of families and the communities they live in, and engage multiple local stakeholders realizing that they are major influences of oral and general health promotion.
